# Preparation and Characterization of a Novel Amidoxime-Modified Polyacrylonitrile/Fly Ash Composite Adsorbent and Its Application to Metal Wastewater Treatment

**DOI:** 10.3390/ijerph19020856

**Published:** 2022-01-13

**Authors:** Yan Sun, Xiaojun Song, Jing Ma, Haochen Yu, Gangjun Liu, Fu Chen

**Affiliations:** 1 School of Public Administration, Hohai University, Nanjing 210098, China; suny@hhu.edu.cn (Y.S.); songxiaojun@hhu.edu.cn (X.S.); jingma2013@cumt.edu.cn (J.M.); 2 School of Public Policy, China University of Mining and Technology, Xuzhou 221043, China; haochen.yu@cumt.edu.cn; 3 Geospatial Science, School of Science, STEM College, RMIT University, Melbourne 3000, Australia; gang-jun.liu@rmit.edu.au

**Keywords:** amidoxime-modified polyacrylonitrile/fly ash composite, fly ash, Zn^2+^, adsorption

## Abstract

The polyacrylonitrile/fly ash composite was synthesized through solution polymerization and was modified with NH_2_OH·HCl. The amidoxime-modified polyacrylonitrile/fly ash composite demonstrated excellent adsorption capacity for Zn^2+^ in an aqueous medium. Fourier transform-Infrared spectroscopy, thermogravimetric analysis, nitrogen adsorption, X-ray diffraction, and scanning electron microscopy were used to characterize the prepared materials. The results showed that the resulting amidoxime-modified polyacrylonitrile/fly ash composite was able to effectively remove Zn^2+^ at pH 4–6. Adsorption of Zn^2+^ was hindered by the coexisting cations. The adsorption kinetics of Zn^2+^ by Zn^2+^ followed the pseudo-second order kinetic model. The adsorption process also satisfactorily fit the Langmuir model, and the adsorption process was mainly single layer. The Gibbs free energy ΔG^0^, ΔH^0^, and ΔS^0^ were negative, indicating the adsorption was a spontaneous, exothermic, and high degree of order in solution system.

## 1. Introduction

Zinc is an important trace element in the human body and one of the heavy metal elements harmful to nature [1,2,3]. The harm of zinc to the human body mainly comes from the accumulation of industrial wastewater in water and organisms, which is a potential threat to human health through the food chain [4,5,6]. With the rapid development of industry, Zn^2+^ ions enter the environment in different ways. Therefore, it is urgent to treat the wastewater containing Zn^2+^ ions and solve the toxicity caused by Zn^2+^ ions [7,8,9,10].

At present, there are many industrial methods available for treating Zn^2+^ in heavy metal wastewater such as chemical precipitation [11], chemical redox [12], filtration [13], ion-exchange [14,15,16], electrolysis [17], evaporation recovery [18], and adsorption [19,20,21,22,23]. Among them, the adsorption method has the advantages of simple operation, high selectivity, and high purification. The materials used to adsorb Zn^2+^ ions are activated carbon [24,25,26,27,28,29], zeolite [30,31,32], chitosan [33,34,35], etc.

Fly ash is a kind of industrial byproduct produced by coal combustion, which is a porous material with a loose structure and uniform pore size distribution [36,37]. Fly ash does great harm to the human ecological environment [38]. Furthermore, the amount of fly ash increases with the increase in thermal power generation. Therefore, fly ash should be utilized. In terms of the concept of treating waste with waste, fly ash as an adsorbent has the advantages of easy access, high treatment efficiency, and low cost [39]. However, it is difficult to separate the solid and liquid after adsorption when fly ash is used directly. The fly ash has a good adsorption basis, and different treatments of fly ash by relevant methods can give it a higher adsorption efficiency for the target adsorbent. The modification of materials is generally based on structural modification, remodeling of internal pore structure, and surface modification of materials. Among them, structural modification changed the original components of the material, and the modification of internal pore structure mainly changed the size of the internal pores and specific surface area and can change its ion exchange performance. Surface modification referred to the surface treatment of the material, adhesion, or adhesion of other compounds according to the intermolecular affinity. Current research on fly ash showed that the more common modification methods of fly ash include acid–base modification, thermal modification, inorganic salt modification, and organic modification. Deng et al. studied the adsorption characteristics of fly ash modified by microwave-assisted alkali on hexavalent chromium. After microwave assisted modification, the adsorption performance of fly ash on hexavalent chromium was improved about two times, and the adsorption process was single molecule adsorption [40]. Oyehan et al. studied the adsorption capacity of mesoporous fly ash grafted with an ultrathin film of polydiallyldimethyl ammonium for phenol in aqueous solution and found that the particle size of fly ash was related to adsorption. The smaller the particle size was, the larger the specific surface area was, and the saturated adsorption capacity of fly ash for phenol in aqueous solution was 13.05 mg/g [41]. Tang et al. studied the adsorption and removal capacity of fly ash for Zn^2+^ and Cu^2+^ ions in aqueous solution [42]. Hui et al. reported that pure and chamfered-edge zeolite 4A prepared from coal fly ash were effective in removing mixed heavy metal ions for the recycling of fly ash [43]. There are some reports about polymer-modified fly ash used in the field of wastewater treatment. Jiang and Liu [43] reported that copolymer of acrylic acid (AA) and acrylamide (AM) was grafted onto the surface of the γ-methacryloxypropyl trimethoxy silane (KH-570) modified fly ash by the inverse suspension polymerization process. A novel magnetic fly ash/poly (acrylic acid) (FA/PAA) composite microgel was prepared for adsorption of Pb^2+^ [44].

Chelating fiber as a high-performance adsorption material has been widely used in many fields such as wastewater treatment [45] and heavy metal ion separation, enrichment, and recovery analysis [46]. Chelating fiber has the advantages of large specific surface area, good adsorption performance, and fast adsorption and desorption rate. In this work, amidoxime-modified polyacrylonitrile/fly ash composite (AO-PAN/FA) was prepared and the adsorption capacity for Zn^2+^ was investigated in a water solution.

## 2. Materials and Methods

### 2.1. Materials

FA was obtained from Huaneng Hunan Yueyang Power Generation Co., Ltd., Yueyang, China. The FA was treated with 0.10 mol/L HCl solution for 24 h and then washed and dried at 100 °C for 24 h.

Acrylonitrile, N,N-methylenebisacrylamide, hydroxylamine (NH_2_OH·HCl), ammonium persulfate (APS), Triton X-100, Na_2_CO_3_, and ZnCl_2_ were of analytical grade obtained from Tianjin Kaixin Chemical Industry Co., Ltd., Tianjin, China. Distilled water was used throughout the experiments.

### 2.2. Preparation of Polyacrylonitrile/Fly Ash Composite

The FA pretreated with HCl was ultrasonically treated with water at a ratio of 1:5. Then, a certain amount of Triton X-100 was added and sonicated for 2 h. Then, acrylonitrile and N, N-methylenebisacrylamide were added into the emulsion, and the mixture was sonicated at room temperature for 0.5 h again, followed by adding APS. The mixture was stirred at room temperature for 0.5 h and then at 60 °C for 3 h under N_2_ atmosphere. The polyacrylonitrile/fly ash composite was obtained, which was washed with distilled water and ethanol in turn, extracted in water for 48 h with Soxhlet extractor and dried.

### 2.3. Preparation of Amidoxime-Modified Polyacrylonitrile/Fly Ash Composite

Next, 0.5 g polyacrylonitrile/fly ash composite was added into 150 mL 20 g/L NH2OH·HCl solution, and then 2 g Na_2_CO_3_ was added. The mixture was heated up to 80 °C and maintained for a period of time. The amidoxime-modified polyacrylonitrile/fly ash composite was collected by filtration and then washed by alcohol.

### 2.4. Characterization

Fourier transform infrared (FT-IR) spectra of samples were measured using Nicolet 370 FT-IR spectrometer (Thermo Nicolet Corporation, American, Madison, WI, USA). Nitrogen adsorption (Brunauer-Elmett-Teller, BET) was performed with a Surface Area and Pore Size Analyzer (Micromeritics TriStar3000) at 77 K/1 bar. Thermogravimetric analysis (TGA) was conducted on a SII TG/DTA 6300 thermogravimetric analyzer. X-ray diffraction (XRD) spectra were collected on an Ultima IV X-ray diffractometer (Japan Science Co., Ltd. Tokyo, Japan) using copper Kα radiation at a voltage of 30 kV and 20 mA over the 2θ range of 5–90°. The morphology was analyzed using a field emission scanning electron microscope (SEM) supplied by ZEISS (Sigma 300, Cambridge, UK). Atomic absorption spectroscopy (AAS, Shimadzuatomic absorption spectrometer, Kyoto, Japan) was employed to analyze Zn^2+^ concentration in the adsorption investigation.

### 2.5. Adsorption of Zn^2+^ in Aqueous Solution

The adsorption experiments were carried out at room temperature. Before the experiments, the standard curve of Zn^2+^ was measured by AAS. Next, 0.2085 g ZnCl_2_ was dissolved in distilled water and diluted with distilled water to volume to 1 L. 40 mL of 100 mg/L Zn^2+^ solution was placed in a 200 mL conical flask. Then 0.1 g of amidoxime-modified polyacrylonitrile/fly ash composite was added to this solution. The absorbance of the supernatant was measured at intervals. The adsorption experiments at different pH values were carried out under the above conditions. The initial pH of Zn^2+^ solution was adjusted in the range of 2–7. The adsorption capacity (*q_e_*) was obtained according to the equation:

(1) $$q_{e}=\frac{\left(C_{0}-C_{e}\right)V}{m}$$

where *C*_0_ and *C_e_* (mol/L) are the initial and equilibrium concentrations of Zn^2+^ ions, respectively; *V* (L) is the volume of Zn^2+^ solution; and *m* (g) is the mass of amidoxime-modified polyacrylonitrile/fly ash composite.

#### 2.5.1. Effect of pH

Next, 40 mL of 100 mg/L Zn^2+^ solution was placed in a 200 mL conical flask and 0.1 g amidoxime-modified polyacrylonitrile/fly ash composite was added. The pH of solution was adjusted to be 2, 3, 4, 5, 6, and 7, respectively. The adsorption proceeded under a constant temperature for a period of time and then was filtered.

#### 2.5.2. Dynamic Test

Next, 40 mL of 100 mg/L Zn^2+^ solution was placed in a 200 mL conical flask and 0.1 g amidoxime-modified polyacrylonitrile/fly ash composite was added. The pH of solution was adjusted to be 6. The adsorption proceeded under a constant temperature with a different adsorption time.

#### 2.5.3. Effect of Temperature

Next, 40 mL of 100 mg/L Zn^2+^ solution was placed in a 200 mL conical flask, and 0.1 g amido-ime-modified polyacrylonitrile/fly ash composite was added. The pH of solution was adjusted to be 6. The adsorption proceeded under a constant temperature with a different temperature.

## 3. Results and Discussions

### 3.1. Characterization of Amidoxime-Modified Polyacrylonitrile/Fly Ash Composite

**Figure 1:**
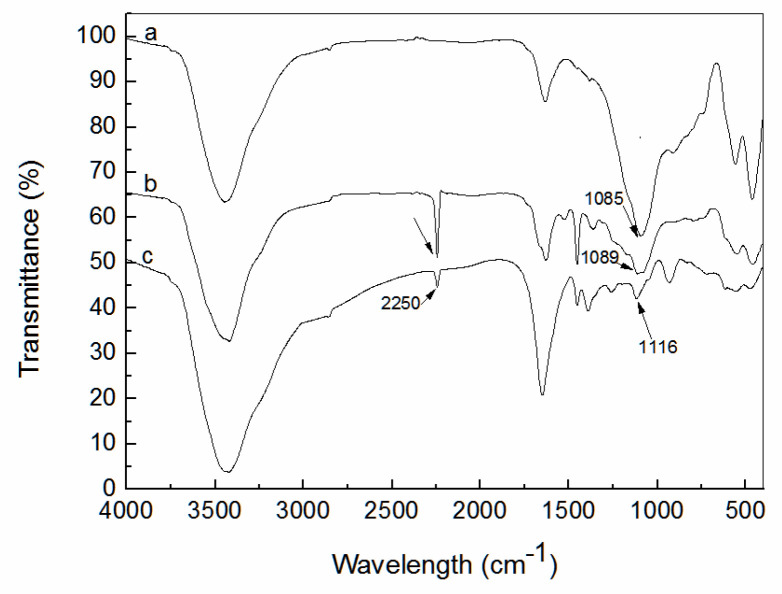
FT-IR spectra of FA pretreated with HCl (a), polyacrylonitrile/fly ash composite (b), and amidoxime-modified polyacrylonitrile/fly ash composite (c).

Figure 1 shows the FT-IR of different composites. The main component of fly ash is silica. As shown in Figure 1, the peaks at 1085 cm^−1^ (a in Figure 1), 1089 cm^−1^ (b in Figure 1), and 1116 cm^−1^ (c in Figure 1) correspond to Si-O coming from fly ash. The peak at 3450 cm^−1^ is attributed to -OH. The peak at 2250 cm^−1^ (b and c in Figure 1) is ascribed to –CN coming from polyacrylonitrile. However, the peak at 2250 cm^−1^ (c in Figure 1) is greatly weakened in comparison with the peak at 2250 cm^−1^ (b in Figure 1).

**Figure 2:**
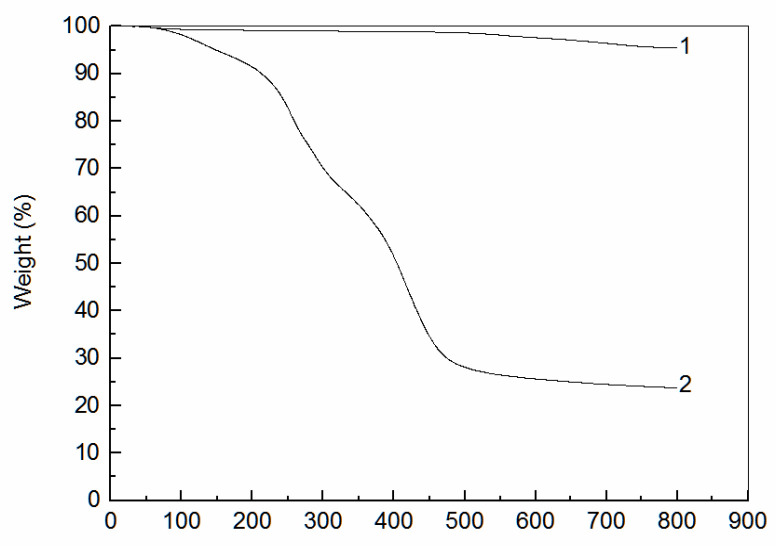
TGA curves of fly ash and amidoxime modified polyacrylonitrile/fly ash composite. (1. Fly Ash; 2. Amidoxime modified polyacrylonitrile/fly ash composite.).

The TGA curves of fly ash and amidoxime modified polyacrylonitrile/fly ash composite are shown in Figure 2. As can be seen, the TGA curve of fly ash had little change. However, the TGA curve of modified fly ash can be divided into two stages. The first stage occurred between room temperature and 100 °C. The free water in amidoxime modified polyacrylonitrile/fly ash composite changed into steam with the increase in temperature. The second stage occurred between 100 and 450 °C, the chemical bonds in the amidoxime modified polyacrylonitrile/fly ash composite were broken, and the structure was destroyed with the increase in temperature to turn into small molecules to form some small organic molecules, such as CH_4_ and CO_2_. The weight loss rate of the amidoxime modified polyacrylonitrile/fly ash composite was 76.28%.

**Figure 3:**
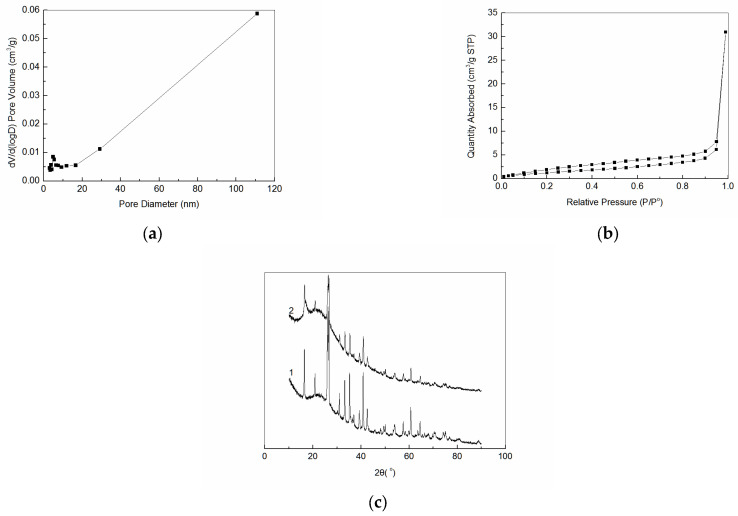
(**a**) N_2_ adsorption-desorption curves of amidoxime-modified polyacrylonitrile/fly ash composite; (**b**) Pore size distribution of amidoxime-modified polyacrylonitrile/fly ash composite; (**c**) XRD curves of fly ash and amidoxime modified polyacrylonitrile/fly ash composite. (1. Fly Ash; 2. Amidoxime modified polyacrylonitrile/fly ash composite.).

**Figure 4:**
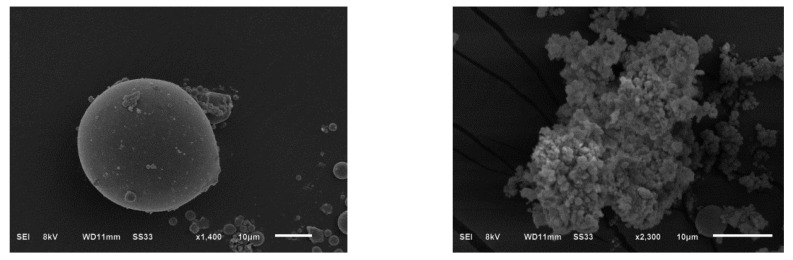
Particle morphology of fly ash (**left**) and amidoxime modified polyacrylonitrile/fly ash composite (**right**) demonstrated by SEM graphs.

N_2_ adsorption–desorption curves and pore size distribution of amidoxime-modified polyacrylonitrile/fly ash composite are shown in Figure 3. As can be seen from Figure 3a, N_2_ desorption adsorption curves belonged to a type IV isothermal adsorption curve. Figure 3b shows that the sample was rich in pore structure. Moreover, the pore size was mainly distributed below 10 nm. The specific surface area calculated by BET was 4.653 m^3^/g. The total pore volume of single point adsorption calculated by BJH was 0.048 cm^3^/g, and the average pore size was 4.862 nm. The XRD spectra of fly ash and amidoxime modified polyacrylonitrile/fly ash composite are shown in Figure 3c. As shown, the XRD peak of fly ash was sharp. However, the XRD peak intensity of amidoxime modified polyacrylonitrile/fly ash composite decreased. SEM graphs of fly ash and amidoxime modified polyacrylonitrile/fly ash composite are shown in Figure 4. The fly ash (left) particles were spherical, and the surface of fly ash showed relatively uniform and smooth characteristics. The surface of amidoxime modified polyacrylonitrile/fly ash composite (right) became very rough, accompanied by cracks, pits, and other phenomena. Many microporous structures were also found on its surface. This kind of irregular folded lamella was caused by the modification.

### 3.2. Effect of Initial pH on Adsorption

**Figure 5:**
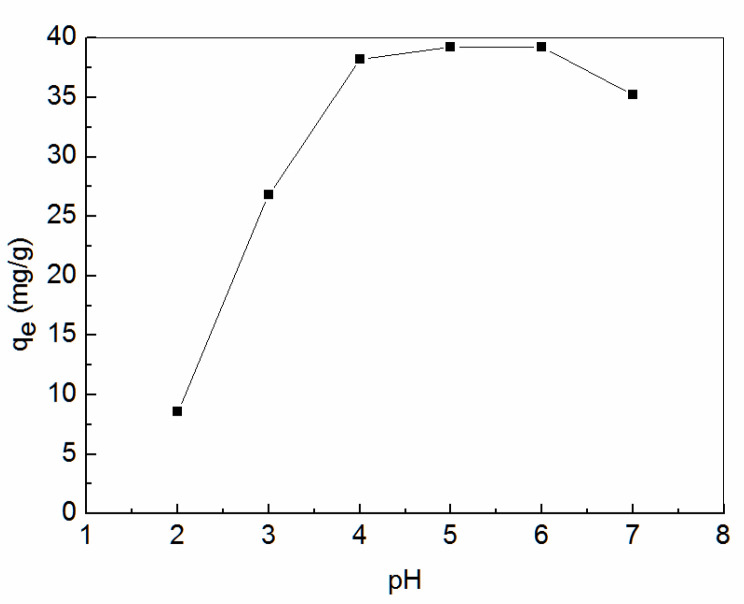
Effect of initial solution pH on equilibrium adsorption capacity (*q_e_*).

The effect of the initial solution pH on *q_e_* was investigated. The result is shown in Figure 5. The *q_e_* increased when the initial pH increased from 2 to 4. However, the increase in adsorption capacity slowed down when the initial pH exceeded 4. The *q_e_* decreased when the initial pH exceeded 6. The *q_e_* of Zn^2+^ is related to its existing form in solution. The large amount of H^+^ in aqueous medium made the functional groups on the surface of amidoxime-modified polyacrylonitrile/fly ash composite protonated at pH < 4. The adsorbable Zn^2+^ sites decreased on the one hand, on the other hand, H+ competed with Zn^2+^ for the limited adsorption sites on the surface of amidoxime-modified polyacrylonitrile/fly ash composite. As a result, the *q_e_* of Zn^2+^ decreased with the decrease in pH value. When the pH value was in the range of 4 to 6, the surface functional groups of amidoxime-modified polyacrylonitrile/fly ash composite were gradually deprotonated. The *q_e_* of Zn^2+^ also increased. When the pH was greater than 6, the formation of zinc hydroxides on the composite surface was not conducive to the adsorption of Zn^2+^, resulting in reduced *q_e_*.

### 3.3. Effect of Coexisting Ions on Adsorption of Zn^2+^

**Figure 6:**
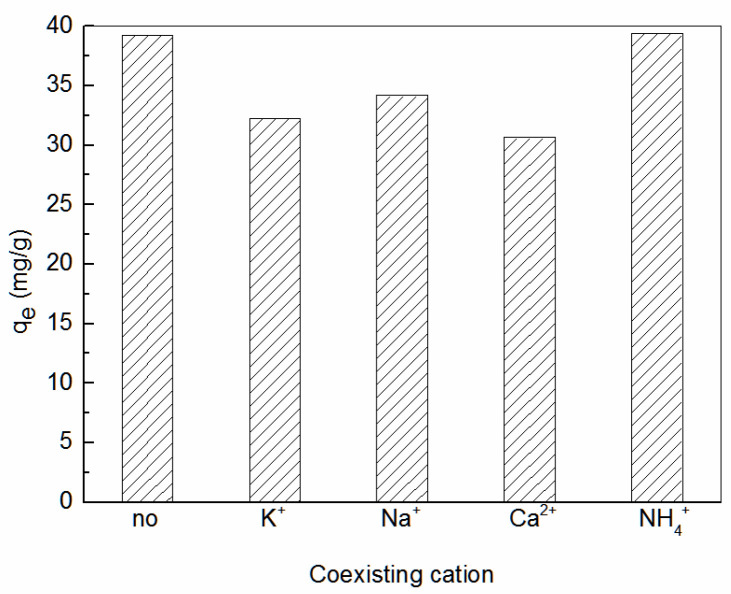
Effect of coexisting anion on the adsorption of Zn^2+^ by amidoxime-modified polyacrylonitrile/fly ash composite.

When the initial concentration of Zn^2+^ C0 was 100 mg·L^−1^, the adsorption temperature was 25 °C, the nitrates of Na^+^, K^+^, and Ca^2+^ were added, respectively, the addition amount was 50 mg·L^−1^. The pH value of solution was adjusted by hydrochloric acid and sodium hydroxide. The influence of cations on the adsorption of Zn^2+^ by amidoxime-modified polyacrylonitrile/fly ash composite was investigated. The experimental results are shown in Figure 6. As can be seen, except for NH_4_^+^, other cations were not conducive to the adsorption of Zn^2+^ by amidoxime-modified polyacrylonitrile/fly ash composite, because coexisting cations competed with the adsorption of Zn^2+^, which reduced the number of adsorption sites on the surface of amidoxime-modified polyacrylonitrile/fly ash composite. Under the condition of weak acid, NH_4_^+^ mainly existed as NH_3_·H_2_O, which had little effect on the adsorption of Zn^2+^ by amidoxime-modified polyacrylonitrile/fly ash composite, but the adsorption capacity of Zn^2+^ slightly increased.

### 3.4. Effect of Contact Time on Adsorption

**Figure 7:**
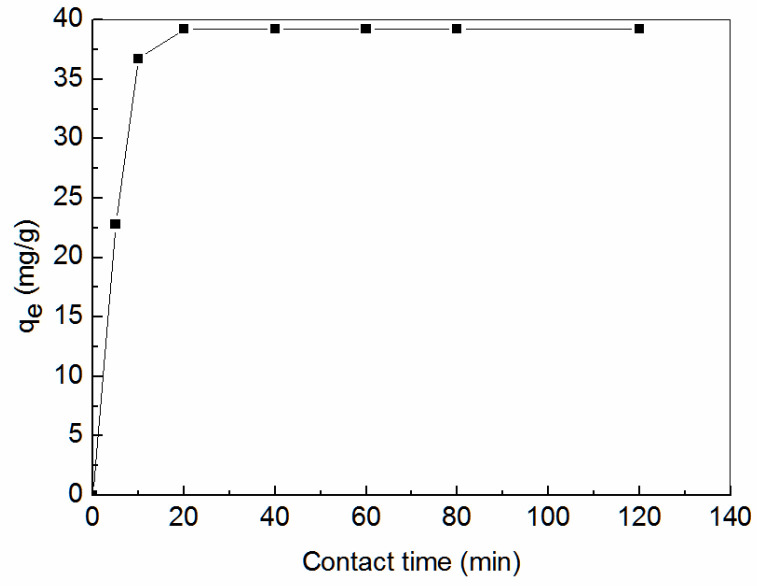
Effect of contact time on equilibrium adsorption capacity (*q_e_*).

Figure 7 shows the effect of adsorption time on the *q_e_* of Zn^2+^ by amidoxime-modified polyacrylonitrile/fly ash composite. As can be seen, the adsorption process of Zn^2+^ by adsorbent can be roughly divided into three stages: fast stage, dynamic equilibrium stage, and slow stage. The *q_e_* increased gradually within the initial 20 min and then tended to be stable with the extension of adsorption time. There were several adsorption sites on the surface of the adsorbent at the beginning of adsorption. It is also beneficial for the Zn^2+^ to enter into the particle through the pores. As the adsorption proceeded, the available sites on the surface of amidoxime-modified polyacrylonitrile/fly ash composite were exhausted. The adsorption rate of Zn^2+^ was manipulated by the rate at which Zn^2+^ was absorbed from the outer to the inner adsorbable sites of amidoxime-modified polyacrylonitrile/fly ash composite. The adsorption rate slowed. To ensure the establishment of the adsorption equilibrium, the experimental adsorption vibration time was set to be 120 min.

### 3.5. Adsorption Kinetics

The adsorption data were fitted by the pseudo-first order kinetic model (Equation (1)) [47], pseudo-second order kinetic model (Equation (2)) [48], and the Elovich equation (Equation (3)) [49], respectively.

(2) $$\ln(q_{e}-q_{t})=\ln q_{e}-k_{1}t$$

(3) $$\frac{t}{q_{t}}=\frac{1}{k_{2}q_{e}^{2}}+\frac{t}{q_{e}}$$

(4) $$q_{t}=\frac{1}{\beta }\ln t+\frac{1}{\beta }\left(\alpha \beta \right)$$

where *q_e_* and *q_t_* (mg/g) are the amount of adsorbate adsorbed at equilibrium and at any time *t* (min), respectively; *k*_1_ and *k*_2_ are the pseudo-first order kinetic adsorption rate constant (min^−1^) and the pseudo-second order kinetic adsorption rate constant [g/(min·mg)], respectively; t is the adsorption time (min); and *α* and *β* are the parameters of the adsorption kinetic model.

**Figure 8:**
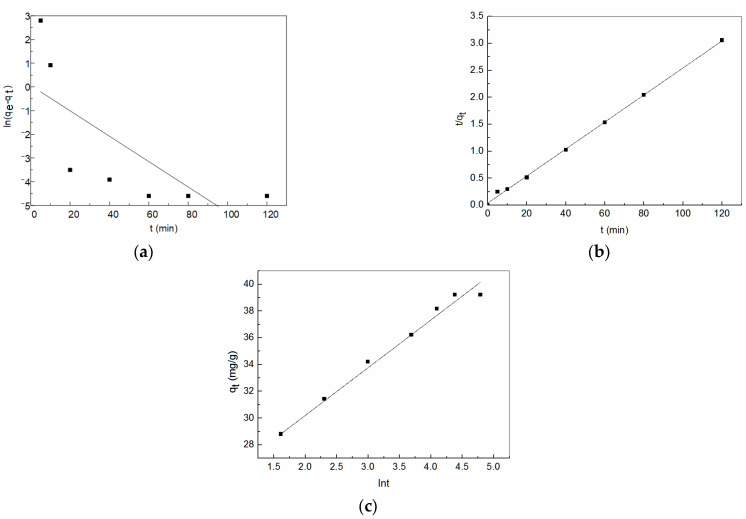
(**a**) Linear fit of pseudo-first order equation; (**b**) Linear fit of pseudo-second order equation; and (**c**) Linear fit of Elovich equation.

Three models were used to fit the dynamic data of amidoxime-modified polyacrylonitrile/fly ash composite, and the fitting results and corresponding parameters are shown in Figure 8 and Table 1. It was found that the adsorption process of Zn^2+^ by amidoxime-modified polyacrylonitrile/fly ash composite was more in line with the pseudo-second-order model, and the correlation coefficients R^2^ were all above 0.99. Moreover, the experimental value (*q_e_*,_exp_) and the theoretical value (*q_e_*,_cal_) differed little. The results indicate that the adsorption of Zn^2+^ on amidoxime-modified polyacrylonitrile/fly ash composite involved the diffusion of the outer liquid membrane and surface adsorption. Furthermore, the parameter α of the Elovich equation was much larger than β, indicating that the initial adsorption rate was very fast, which was consistent with the experimental results.

### 3.6. Adsorption Isotherm

The isothermal adsorption curve refers to the relationship between the concentrations of solute molecules in two phases when the adsorption process on the interface reaches equilibrium at a certain temperature. The adsorption isotherm is helpful to understand the nature of the adsorption phenomenon, and the adsorption capacity of amidoxime-modified polyacrylonitrile/fly ash composite at a specific concentration of Zn^2+^ can be calculated by the isotherm adsorption model.

The linearized Langmuir [50] and Freundlich [51] isotherms are given by Equation (5) and Equation (6), respectively.

(5) $$\frac{C_{e}}{q_{e}}\;=\frac{1}{q_{max}b}+\frac{C_{e}}{q_{max}}$$

(6) $$\ln q_{e}=\ln K+\frac{1}{n}\ln C_{e}$$

where *q_e_* is the equilibrium adsorption capacity (mg/g), *C_e_* is the concentration of Zn^2+^ ions in the solution at equilibrium (mol/L), *q*_max_ is the maximum adsorption capacity (mg/g), and *b* is a constant related to the free energy of adsorption. *K* is a constant related to adsorption capacity and adsorption strength; and n is the Freundlich constant.

**Figure 9:**
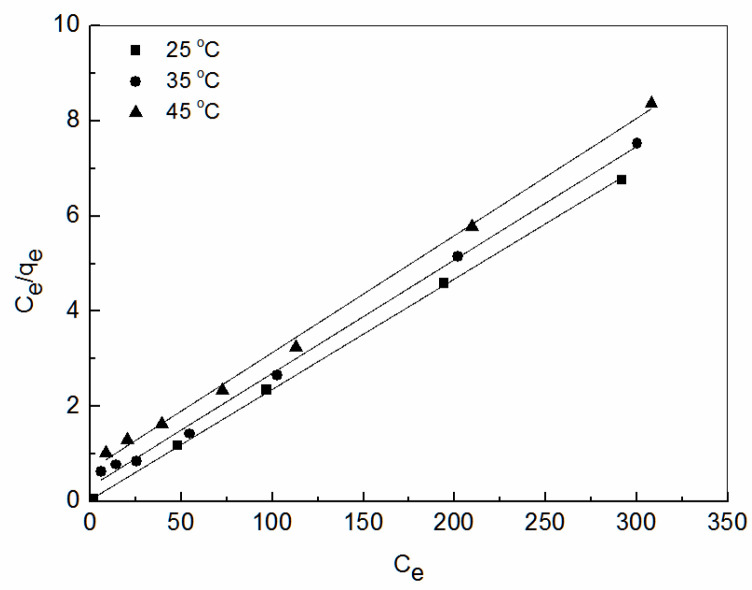
Linearized Langmuir isotherms obtained from Zn^2+^ adsorption on amidoxime-modified polyacrylonitrile/fly ash composite.

The Langmuir and Freundlich models were used to fit the adsorption data of Zn^2+^ at 25 °C, 35 °C, and 45 °C. The fitting results are shown in Figure 9 and Table 2. As shown, the coefficient of correlation R^2^ of Langmuir model was greater than that of the Freundlich model, indicating that the adsorption data were more consistent with the Langmuir isothermal adsorption model. The Langmuir isothermal adsorption equation assumes that the adsorption is monolayer, solute and solvent molecules have approximately the same volume or the same adsorption site; the adsorption of solute is regarded as the result of the exchange between solute molecules in the solution and the adsorbed solvent molecules in the adsorption layer. Table 2 summarizes some of the recent results of Zn^2+^ removal from wastewater using fly ash.

According to the Langmuir theory, it is speculated that Zn^2+^ is uniformly adsorbed on the active site (NH_2_-C = N-OH) on the surface of amidoxime-modified polyacrylonitrile/fly ash composite. When all the active sites are adsorbed on Zn^2+^, the adsorption amount reaches saturation, and the adsorption is in equilibrium. In Table 3, both the Langmuir adsorption constant b and the adsorption capacity qmax decreased with the increase in temperature, indicating that the temperature rise was not conducive to the adsorption process.

The essential characteristic of the Langmuir isothermal adsorption is that it can be expressed as a dimensionless constant *P_L_*. It can characterize the adsorption performance, predicting the binding force of adsorbent and adsorbent:

(7) $$P_{L}=\frac{1}{1+bC_{0}}$$

where *P_L_* is the Langmuir isothermal adsorption constant and *C*_0_ is the initial concentration of the solution (mol/L).

When 0 < *P_L_* <1, adsorption is easy under the experimental conditions studied. The initial concentration at each temperature was selected, and *P_L_* was calculated to be 0.004~0.0051, 0.038~0.3043, and 0.0639~0.4766, respectively. It can be seen that *P_L_* is between 0 and 1, and *P_L_* decreased with the increase in Zn^2+^ concentration at the same temperature, indicating that the increase in the initial concentration of Zn^2+^ in the adsorption solution was conducive to the adsorption, and adsorption was favorable under the whole test conditions.

### 3.7. Thermodynamics

The direction and difficulty of the adsorption reaction can be judged by the thermodynamic parameters such as Gibbs free energy change Δ*G*, enthalpy change Δ*H*, and entropy change Δ*S*.

The Vant Hoff equation is calculated as follows:

(8) $$\Delta G^{0}=\ln b$$

(9) $$\ln b=\frac{\Delta H^{0}}{RT}\;\frac{\Delta S^{0}}{R}$$

(10) $$b=q_{e}/C_{e}$$

where *R* is the gas constant [8.314 J/(mol·K)]; *T* is temperature (*K*); *b* is Langmuir isothermal adsorption constant; and Δ*H* and Δ*S* can be obtained from the slope and intercept of ln*b* and 1000/T, respectively.

**Figure 10:**
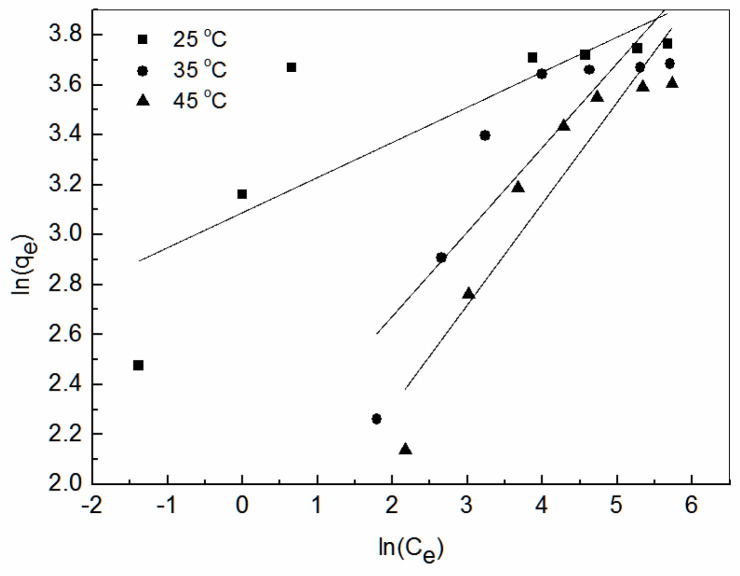
Linearized Freundlich isotherms obtained from Zn^2+^ adsorption on amidoxime-modified polyacrylonitrile/fly ash composite.

The fitting results are listed in Table 4. The negative value of Δ*H*^0^ indicated that the adsorption reaction of Zn^2+^ was exothermic. Adsorption Gibbs free energy Δ*G*^0^ is the embodiment of the adsorption driving force and adsorption preference, and Δ*G*^0^ was negative, which indicated that the adsorption process of Zn^2+^ was spontaneous. Furthermore, Δ*G*^0^ increased with the increase in temperature, indicating that the reaction was more easily carried out at low temperature (Figure 10).

Entropy change Δ*S*^0^ is the algebraic sum of entropy change of the whole system process, which reflects the change of the chaos degree of the existing state in the system. The Δ*S*^0^ is small and the system is in a relatively ordered state. The Δ*S*^0^ is large and the system is in a relatively disordered state. Δ*S*^0^ is negative, indicating that the order degree of the whole solution system is improved through the adsorption of Zn^2+^ by the adsorbent. According to the exchange theory, absorption for solid–liquid exchange adsorption, solute molecules from solution phase adsorption exchange to lose part of solid-liquid interface degrees of freedom, this is a process of entropy, at the same time, on the adsorbent adsorption Zn^2+^, to a large number of water molecules on the adsorbent desorption, the original on the surface of the adsorbent and tidy, compact arrangement of water molecules desorption to freedom of movement was a process of increasing entropy. The entropy change of the whole system is the sum of the entropy changes of the above two processes, and its value is negative, indicating that the order of the whole system has been enhanced.

## 4. Conclusions

In this study, amidoxime-modified polyacrylonitrile/fly ash composite was prepared. The *q_e_* of Zn^2+^ can be increased by decreasing temperature and increasing pH and initial concentration of Zn^2+^. The pseudo-second order kinetic equation can better describe the adsorption process. The adsorption of Zn^2+^ on the composite was dominated by chemical adsorption. The negative Δ*G*^0^ and positive ΔH^0^ indicate that the adsorption process is endothermic and spontaneous. The treatment of adsorption of Zn^2+^ by amidoxime-modified polyacrylonitrile/fly ash composite has the advantages of simple operation, mild adsorption conditions, almost no additional energy and power consumption, no introduction of secondary pollution. This was not only a “green” method saving energy but also in line with the concept of environmental protection.

## Figures and Tables

**Table 1:** Kinetic constants for adsorption of Zn^2+^ ions.

Kinetic Model	Pseudo-First Order Kinetic			Pseudo-Second Order Kinetic			Elovich Equation		
*q_e_*_,exp_ (mg/g)	*k*_1_ (min^−1^)	*q_e_*_,cal_ (mg/g)	R^2^	*k*_2_ g/(min·mg)	*q_e_*_,cal_ (mg/g)	R^2^	*α*	*β*	R^2^
39.81	0.0536	1.0644	0.4453	0.0250	39.92	0.9985	2297	0.2803	0.9785

**Table 2:** Summary of Zn^2+^ adsorption on fly ash according to references and present study.

Adsorbent	Adsorption Capacity (mg/g)	Reference
ZIF-8/fly ash composite	197	[52]
lime activated fly ash	33.13	[53]
fly ash-based geopolymer	35.18	[54]
modified fly ash	13.38	[55]
amidoxime-modified polyacrylonitrile/fly ash composite	43.08	This work

**Table 3:** Equilibrium parameters for adsorption of Zn^2+^.

T (°C)	Langmuir Model			Freundlich Model		
	*q_e_*_,cal_	*b*	R^2^	*K*	*n*	R^2^
25	43.08	0.6090	0.9997	21.96	7.115	0.6189
35	41.93	0.0762	0.9971	7.38	2.961	0.7441
45	40.65	0.0366	0.9970	4.49	2.462	0.8617

**Table 4:** Thermodynamic parameters for adsorption of Zn^2+^.

T (°C)	*b*	Δ*G*^0^ (kJ/mol)	Δ*H*^0^ (kJ/mol)	Δ*S*^0^ (J/(mol·K))
25	609	−15.885	−116.88	−334.27
35	76.2	−11.096		
45	36.6	−9.518		

## Data Availability

Not applicable.
